# A patient-centred approach to health service delivery: improving health outcomes for people with chronic illness

**DOI:** 10.1186/1472-6963-13-251

**Published:** 2013-07-03

**Authors:** Masoud Mirzaei, Clive Aspin, Beverley Essue, Yun-Hee Jeon, Paul Dugdale, Tim Usherwood, Stephen Leeder

**Affiliations:** 1 Yazd Cardiovascular Research Centre, Shahid Sadoughi University of Medical Sciences, Yazd, Iran; 2 Menzies Centre for Health Policy, University of Sydney, Sydney, Australia; 3 Sydney Nursing School, University of Sydney, Sydney, Australia; 4 Centre for Health Stewardship, College of Medicine and Health Sciences, Australian National University, Canberra, Australia; 5 Discipline of General Practice, University of Sydney, Sydney, Australia; 6 The George Institute for Global Health, Sydney, Australia; 7 Poche Centre for Indigenous Health, University of Sydney, Sydney, Australia

## Abstract

**Background:**

The Wagner Model provides a framework that can help to facilitate health system transition towards a chronic care oriented model. Drawing on elements of this framework as well as health policy related to patient centred care, we describe the health needs of patients with chronic illness and compare these with services which should ideally be provided by a patient-centred health system. This paper aims to increase understanding of the challenges faced by chronically ill patients and family carers in relation to their experiences with the health care system and health service providers.

**Method:**

We interviewed patients, carers and health care professionals (HCPs) about the challenges faced by people living with complicated diabetes, chronic heart failure or chronic obstructive pulmonary disease.

**Results:**

Patients indicated that they had a range of concerns related to the quality of health care encounters with health care professionals (HCPs), with these concerns being expressed as needs or wants. These included: 1) the need for improved communication and information delivery on the part of HCPs; 2) well organised health services and reduced waiting times to see HCPs; 3) help with self care; 4) greater recognition among professionals of the need for holistic and continuing care; and 5) inclusion of patients and carers in the decision making processes.

**Conclusions:**

In order to address the challenges faced by people with chronic illness, health policy must be more closely aligned with the identified needs and wants of people affected by chronic illness than is currently the case.

## Background

Chronic, non-communicable diseases are currently responsible for around 70% of the total burden of illness and injury experienced by the Australian population and this proportion is expected to increase to almost 80% by 2020 [1]. According to the most recent burden of disease study in Australia (2003), diabetes, cardiovascular disease and chronic respiratory diseases are the top three conditions responsible for the majority of Disability Adjusted Life Years (DALYs) lost in males. In females, these conditions are among the top seven causes of DALYs lost [2]. Among other things, the aging population and an increase in some risk factors among young population are likely to contribute to increased mortality and morbidity in the near future in developed countries including Australia [3].

In 1999-2000, 17 per cent of hospital admissions were for the chronic illnesses of respiratory and cardiovascular disease, diabetes and cancer. These admissions accounted for 36 per cent of total public hospital bed days. The cost of providing hospital care for people with respiratory and cardiovascular disease, diabetes and cancer, was estimated at $1.1 billion in New South Wales (NSW) in 1999-2000 [4].

Wagner has developed a chronic care model to address issues such as these and to facilitate health system transition towards a chronic care oriented model [5-7]. The model identifies patient centeredness, effectiveness, efficiency, equity [7] and timeliness as essential elements of efficient health service delivery for people with chronic illness.

Patient centredness in health care has been shown to increase patient engagement, satisfaction and compliance, improve quality of life and reduce patient anxiety [8,9]. Observational studies have demonstrated that patients prefer patient centred care, and those who receive it report enhanced health outcomes [10]. A review by Coulter and Ellins concluded that strategies for informing, educating, attending to needs and wants and involving patients in their chronic care management, all of which are essential ingredients of patient centredness, are effective [11].

Drawing inspiration from the Wagner chronic care model, the Serious and Continuing Illness Policy and Practice Study (SCIPPS) investigated the role of patient-centredness in improving health services for people with chronic illness. SCIPPS is a multi-staged study designed to improve the management and care of people with chronic illness including chronic heart failure (CHF), type 2 diabetes and chronic and obstructive pulmonary disease (COPD) in the Australian Capital Territory (ACT) and NSW. These three index conditions were chosen because they are among the main causes of DALYs lost. As well, they are costly and continuing illnesses that utilise multiple health services, treatments and technologies. Furthermore, a wide spectrum of primary and specialist services, acute and ambulatory care, and community support services, as well as effective self care, is needed for their successful management.

As part of SCIPPS, a qualitative study was undertaken. Patients and carers were interviewed about their experiences of living with the index conditions, including their experiences with the health system and health service providers. Overall findings from the study have been reported elsewhere [12,13].

The aim of this paper is to explore and describe the experiences of patients with chronic illness and their carers, with a particular focus on the challenges that patients confront when interacting with the health system and health service providers.

## Methods

This study used purposive sampling to recruit participants in order to collect data through in-depth interviews and focus groups with patients (N = 52), their carers (N = 14) and health care professionals (N = 63).^a^ The characteristics of patients and carers summarised in Table 1 and those of HCPs summarised in Table 2. Participants were recruited from two locations, the Australian Capital Territory (ACT) and Sydney West, New South Wales (NSW).

**Table 1:** Characteristics of patients and carers

**Categories**	**Sub-categories**	**Patient (N = 52)**	^**§**^**Carer (N = 14)**
Residence	ACT	26 (50.0%)	6 (42.9%)
	Sydney West	26 (50.0%)	8 (57.1%)
Gender	Male	28 (53.8%)	1 (07.1%)
	Female	24 (46.2%)	13 (92.9%)
Age	Less than 45 yrs	^**^**^1 (1.9%)	2 (14.3%)
	45-64 yrs	16 (30.8%)	5 (35.7%)
	65-85 yrs	35 (67.3%)	7 (50.0%)
Culturally and linguistically diverse (CALD)	CALD background	11 (21.2%)	5 (35.7%)
Indigenous	Indigenous background	7 (13.5%)	0
Finance	Experiencing financial difficulties	31 (59.6%)	11 (78.6%)
Marital Status	Married/de facto/living with a partner	29 (55.8%)	13 (92.9%)
Work Status	Employed	5 (9.6%)	2 (14.3%)
	Not employed	47 (90.4%)	12 (85.7%)
Diagnosis	Type 2 diabetes	16 (30.8%)	^*^4 (28.6%)
	CHF	15 (28.8%)	^*^2 (14.3%)
	COPD	10 (19.2%)	^*^5 (35.7%)
	More than one index condition	11 (21.2%)	^*^3 (21.3%)
	Average length of illness	16.5 years	^*^21.4 years
	Other co-morbid conditions	43 (86.5%)	^*^11 (85.7%)
Visit to GP	Weekly	4 (7.7%)	^*^1 (7.1%)
	Fortnightly	8 (15.4%)	^*^6 (42.9%)
	Monthly	23 (44.2%)	^*^5 (35.7%)
	Bi-monthly	3 (5.8%)	^*^0
	Quarterly	7 (13.5%)	^*^1 (7.1%)
	Half-yearly	2 (3.8%)	^*^0
	Only when necessary	5 (9.6%)	^*^1 (7.1%)
Family Carer	Have a family carer	^#^22 (42.3%)	NA
	Average length of caring	^#^14.7 years	12.5 years
	Relationship of carer to patient		
	Offspring	6 (27.3%)	4 (28.6%)
	Spouse/partner	14 (63.6%)	10 (71.4%)
	Other	2(9.1%)	0
	Carer receives financial assistance	^#^5 (22.7%)	5 (35.7%)
	Carer receives informal support	^#^14 (63.6%)	9 (64.3%)

^§^ Nine carers were spouses or offspring of the patients interviewed**, **^**^**^ One Indigenous participant was younger than 45 years old (given a gap of approximately 20 years shorter life expectancy than non-Indigenous Australian it was deemed to be appropriate to be included in the study), ^*^ Denotes the carer’s account about the patient’s condition and management.

^**#**^ Denotes the patient’s account about the carer.

**Table 2:** Characteristics of health care professionals

**Categories**	**Sub-categories**	**HCP (N = 63)**
Residence	ACT	26 (41%)
	Sydney West	37 (59%)
Gender	Male	19 (30%)
	Female	44 (70%)
Work setting	Hospital (in and out-patient services)	22 (35%)
	Community health service	9 (14%)
	General practice	22 (35%)
	Rehabilitation centre	4 (7%)
	Policy agency	5 (8%)
Time in this employment	Less than 5 years	18 (29%)
	5-10 years	18 (29%)
	More than 10 years	27 (43%)
Employment status	Full time	55 (87%)
	Part time	8 (13%)

Based on a preliminary analysis of the patient and carer interviews, problems associated with health service delivery and health care professionals were identified as key issues. This led to a decision to conduct eight focus group discussions (FGDs) with HCPs. During these FGDs, participants were questioned about what they perceived to be the key barriers and facilitators that patients and carers experience while accessing and utilising relevant health services. While the primary focus of the study was on patient experience, it was considered important to recruit a sizeable number of HCPs in order to ensure that the study gathered the full range of diverse experiences related to the chronic illnesses of interest. FGDs were considered to be more appropriate for data collection from HCPs since these allowed them to generate robust discussion. Individual interviews were considered to be most appropriate for patients and carers since it allowed them to talk more freely about issues of a personal nature.

Participants were recruited through referrals from general practices, local hospitals, community health services, specialist clinics, health care consumer organisations, and Aboriginal Medical Services located in Sydney West and ACT. Eligible participants included patients aged between 45 and 85 with one or more of the three index conditions, as well as family care givers and HCPs who have been involved in providing care for people with the index conditions. This age range was chosen because of the high prevalence rates of chronic illness among people of this age. HCPs were recruited from local hospitals, community health services, consumer organisations and Aboriginal medical services in order to represent the broad range of services from which people with chronic illness access services. A sampling grid was used to ensure that participants of varied age, culture, ethnicity, severity of the illness and health care setting, were included. For FGDs, community and hospital nurses, allied health workers, general practitioners and hospital-based staff were included (see Table 2 for details).

Ethical approval to conduct the study was granted by the University of Sydney Human Research Ethics Committee, the Sydney West Area Health Service Human Research Ethics Committee, the Australian National University Human Research Ethics Committee, and the ACT Health Human Research Ethics Committee. All participants provided informed consent and were given the option of stopping the interview or withdrawing from the study at any point if they chose to.

The in-depth, semi- structured interviews were conducted by members of the research team who were also closely involved in the data analysis and writing of manuscripts. Each interview lasted for between 45 and 90 minutes and was followed by a 10-15 minutes interviewers’ assisted survey. The survey obtained basic information about the participants such as demographics, health care encounters and the clinical and medication profiles [14]. The survey data were descriptively analysed using SPSS version 13.00 (SPSS Inc; Illinois, USA).

Each interview began with a question asking the participant what it is like to live (or care for someone) with chronic illness. The interviewers asked eight consecutive questions followed by probing questions to further explore issues. The main and probing questions are summarized in Table 3.

**Table 3:** Key and probing questions asked during interviews with patients and carers and focus group discussions with health care professionals

**Interviews**	
**Key questions**	**Probing questions**
What have been the greatest challenges that you have faced as a patient OR as a carer?	1) Can you tell me exactly what happened? (tell me more about that), 2) Why do you think that happened? 3) How did that affect you? 4) How do you think it affected people around you? 5) How did you cope? 6) What do you think would prevent a similar thing happening again? 7) I understand that may have been difficult for you, what other challenges have you faced?
What has been your experience with health professionals in terms of managing your diabetes/COPD/CHF OR as a carer.	In all your experiences with health professionals can you think of any experiences that could be improved?
What has been unhelpful about the health professionals who have provided care for you?	1) Have you ever had an interaction with a health professional that has been a problem for you? 2) What could they do that would most improve your care? 3) Who do you think should be involved in making decisions about your treatment and care?
Apart from medical treatment and other professional health care, what else or who else helps you cope with your COPD/CHF/diabetes?	1) Is there any help that you don’t get now that you believe would assist in living with your COPD/CHF/diabetes? 2) Do you think that is something that others with your condition may also appreciate or is that something that might be more specific to your own circumstances? 3) Why is that?
I’m interested in experiences you have had with health services in terms of managing your COPD/CHF/diabetes. - Can you describe some of the helpful and unhelpful aspects of health services that you have received?	1) Can you tell me exactly what happened? 2) Why do you think that happened? 3) How did that affect you/others? 4) How did you cope? 5) What do you think would prevent a similar thing happening again? 6) Can you think of anything you would change in the health service to improve this experience or even to prevent it from happening again? 7) In an ideal world, what would you want the health service to provide for people living with diabetes/COPD/CHF?
**Focus group discussions**	
Key questions	Probing questions
What sort of problems do you face as a health professional in providing care for people with diabetes, COPD and CHF (OR: these three conditions)?	What do you think are the main cause(s) of those problems?
What sort of problems do you think patients and their carers face in managing their condition?	Generally speaking, people we have interviewed indicated that they struggle with managing their chronic conditions; that they find it difficult to do the ‘right things’ on an on-going basis.
	What are your thoughts on that? (Possible prompts: acting on risk factors, warning signs; identifying triggers)
Many people are managing more than one condition at the same time and they are finding it really hard. Can you talk about that for a moment?	
Patients have also told us that managing a chronic illness can cause a financial burden.	What sort of problems do you think this poses for your patients?
We’ve discussed some of the challenges you and your patients face. What changes can you think of that would assist you and your patients in addressing these challenges	
Given the competing demands for resources, what sorts of things should we be investing in to address the chronic disease crisis in Australia?	

Interviews continued to the point that sufficient data had been gathered and interviews were no longer providing new concepts. At this point it was considered that saturation had been reached and no further interviews took place. In the pilot phase of the study, issues relating to health services and health care providers were identified as key concepts in managing chronic illness. These issues were then further explored during FGDs with HCPs.

All interviews and focus group discussions were electronically recorded and transcribed using professional transcription services. All transcripts were entered into NVivo 7, a computerized qualitative data analysis program which was used to manage the data (QSR International Pty Ltd; Doncaster, Australia). Content analysis was used to analyse and make sense of the data. The process involved analysis by topic, and each transcript was segmented by these topics into categories, constructs and domains in order to identify patterns [15]. The interviews and FGDs were managed as one dataset. In the FGDs, it was not possible to identify individual participants so we were not able to ascribe particular comments to individual HCPs or to recognise their organisation.

Data collection and analysis were carried out by the same team of researchers who checked regularly to ensure the accuracy of transcripts and consistency of the qualitative and quantitative data by comparing the interviews and the questionnaires. Field notes and memos were also used to facilitate reflexivity and the interviewers met frequently to ensure the consistency of the processes of interview, data entry, coding and interpretation of the data. Analysis revealed that the dominant themes as reported here were closely related to the concept of patient centred care.

## Results

Most of the patients and care givers were older than 65 years and were not currently working. Most patients had a history of having lived with at least one chronic condition for at least ten years and most had regular contact with their general practitioners (GPs) (Table 1). We identified five major issues related to patient-centred care which limited optimal management of patients with chronic illness and their carers: poor communication with HCPs and the provision of information; poor organisation of service delivery and long waiting times to see HCPs; insufficient facilitation of self care; insufficient holistic and continuing care; and lack of patients and carers involvement in decision making (see Table 4 for details).

**Table 4:** Issues related to health service encounters raised by chronically ill patients, and suggestions of patients, carers and health care professionals for overcoming them

**Challenges raised by participants**	**Patients’ and carers’ suggestions**	**HCP suggestions and solutions**
***•Communication and delivery of information***		
Inadequate explanation of illness, prescribed medication and side effects	HCPs provide patients with a written care plan in plain language	Improvement of health literacy
Confusing and conflicting information provided	Professional organisations endorse guidelines	Better communication between different professional groups
Practical examples of appropriate diets and concepts not provided (e.g., Glycemic Index)	NS	NS
Lectures given by HCPs seem pointless	More interactive learning experiences such as support groups	Improved communication between patients and HCPs
No explanation given about the side effects of medication	NS	Enhanced information technology infrastructure
No help provided in accessing internet	NS	Enhanced information technology infrastructure
CALD participants want information in their own language	GPs increase their awareness of cultural issues and exercise care in providing information to CALD patients	Improved communication between professionals and CALD groups
HCP seen as inflexible and unaccommodating	NS	Need to improve accountability between service providers
Outdated information about disease management	NS	Enhanced information technology infrastructure
Insufficient information about community resources provided	Provide access to relevant data bases	Better co-ordination of care
Poor communication by interns	Improved supervision by specialists before diagnosing conditions with a poor prognosis such as cancer	Need to improve accountability between service providers
***•Organisation of service delivery and waiting time to see HCP’s***		
Arranging appointments in urgent situations	On call doctors to give information and directions	Need to reduce communication gaps
Arranging appointments when new symptoms appear	Electronic booking systems	Enhanced electronic infrastructure
Long waiting time to make appointments and delays in seeing HCPs	Phone or SMS patients to inform them of possible delays	Need to reduce communication gaps
Inflexible appointment times	Greater flexibility	NS
Rigid eligibility criteria which exclude some people from inpatient or outpatient care	Revision of eligibility criteria of services to ensure they cover patients at different states of disease	Need to improve communication between various service providers
***•Facilitation of self care***		
Insufficient support for self-care	NS	NS
Insufficient written information about what to do in different conditions	Handbooks and disease specific information packages	Enhanced communication
Insufficient follow up and monitoring of patient’s condition as well as new symptoms	GP’s and case coordinators follow up, from hospital or private health insurance	Need to improve co-ordination of care by service providers
***•Single illness focus***		
GPs and specialist focus only on the immediate symptoms and conditions	Holistic approach to patient’s conditions with careful review of medical history	Need to improve fragmentation of services
***•Inclusion of patients and carers in decision making***		
Lack of trust in patient’s knowledge and understanding of signs and symptoms	NS	Need to improve health literacy
Patients don’t feel hopeful and motivated about the future	Provide up to date information and review of the latest developments in lay language	Need to improve patients’ focus on management of conditions
Failure to ask patients about their tolerance of available treatments		Be sure to ask patients about any tolerance or compliance issues before prescribing medications

HCP: Health Care Professional, NS: No suggestion, CALD: Culturally and Linguistically Diverse.

### Poor communication practice and provision of information

Almost half of all patients and carers expressed concerns about the relevance and usefulness of the health information that they received from HCPs especially as it related to their condition(s). Participants struggled to understand the material and advice that they were given and this often led to non-compliance. One said,

*A lot of the books written on diabetes are perhaps a little too sophisticated for the ordinary man-in-the-street diabetic. It’s hard for them to understand unless it’s written in very simple language …//… they say you should have a minimum of 15 g of carbohydrates with each meal. Well, how much is that and what is it? People don’t know if it’s one slice of bread or a small potato. Then the GI index…//… people think potato and rice and stuff is low GI, but it’s not.* (75 years old female with diabetes)

Participants suggested ways of overcoming this problem. Many indicated that they wanted materials to be written in lay language and that it should ideally be delivered interactively, e.g., shopping or cooking tours, to learn how to manage their conditions. They wanted to be actively involved in their treatment, by participating in the development of their care plans. As well, they wanted to know more about medications and the side effects that these could have. Online material that could be accessed by them, or another member of their family, would be helpful in overcoming some of these problems. They wanted to be able to access information about the most recent advances in managing their conditions. One option was to have access to training programs or similar facilities so that they could ask about their illness and its acute episodes at the time when they needed answers. For example,

*They also have shopping tours with a dietitian. You’ve got to pay… But they go around like Woolworths* [a chain supermarket] *and she’ll explain the labels on the things and everything.* (64 years old female with diabetes)

*Oh yes, I think it* [interactive learning] *makes a difference…It could be much more communicative, much more pleasant, much more interactive – where it’s just bomb, bomb, bomb straight down the line you know and that’s it.* (53 year old male with diabetes)

These observations were confirmed by HCPs in all eight focus groups who also identified poor communication and information delivery as a major barrier to good health care for chronically ill patients. They linked this observation to patients’ low levels of health literacy, and to conflicting and confusing messages from other HCPs, including specialists who are often busy and lack sufficient time to provide thorough information. They also suggested that patients and carers sometimes hesitated to ask questions of doctors due to doctors’ time constraints, something which they recognised as a feature of the way in which health systems were organised. Some HCPs also felt that some patients experienced difficulties with communication because of confusion caused by information received from the media and the internet.

HCPs felt that health service providers needed to deliver information to patients in a more coordinated and organized manner than was currently the case in many health services.

### Poor organisation of service delivery and long waiting time to see HCPs

Multiple appointments associated with chronic illness management, little flexibility in making and changing appointment times and rigid eligibility criteria for health services often led to a sense of confusion on the part of patients and a lack of follow-up by HCPs. Eighteen patients expressed confusion in following up the many appointments related to their chronic illness. A common outcome patients and carers used to address these problems was to request an ambulance to take them to hospital when in need of urgent treatment or to wait for serious episodes to evolve before seeking help. Five patients (10%) believed that the quickest and cheapest way to get treatment was to be admitted to hospital (see Table 4).

Making appointments to see HCPs is difficult for people with chronic illnesses, but it can be especially difficult for people in paid employment. Patients also found it difficult to plan ahead and organise services well in advance of a serious episode of their condition. Often there can be a long waiting periods to schedule an appointment as well as delays in seeing the doctor at the appointment time, which can affect working time. An end result for many patients facing these barriers is often a loss of productivity and income.

*But then - and that’s what stops you too with going to work because these clinics are on Wednesdays. I mean you can go in during your lunchtime, but then there are other people before you so therefore you’ve got to take a day off to really have the time and wait..//.. you need the time off. And it’s rushed. It’s not the times - I work part-time, but they’re not the times that I have off.* (54 years old female with diabetes)

HCPs acknowledged that they had difficulties in organising services and that this sometimes resulted in delayed waiting times to see HCPs. They worried that it was difficult to connect a specialist team to patients unless there was a crisis.

### Insufficient facilitation of self care

Fifteen patients and carers said that they had problems with self care and that they needed more support from the health and aged care systems than it currently provided in order to manage their chronic illness effectively. Examples given by participants to overcome this included: telephone hotlines where a HCP would be available to answer urgent questions and provide updated information about the latest progress in disease management as well as handbooks that address basic facts about each illness and its management. They also wanted to know the practical and clear boundaries and limits of their conditions so that they could manage their illness with greater confidence in their day to day life. They also expressed the need for self care support for changing their behaviours and lifestyle.

While most patients recognised that close monitoring of their condition by their GP could occasionally avoid hospital admission, they wanted to avoid having to consult their GPs every time something went wrong or their condition changed in ways that they did not understand. In contrast, those patients who had private health insurance appreciated help from their insurer in providing allied health services and treatment plans. Patients also recognised the value of close monitoring of their condition by their general practitioner. For example one said:

[My GP] *tells me what the boundaries are. Tells me what the parameters are. Goes beyond, goes beyond the minimum, he’s* [GP] *not abrupt and authoritarian and demanding.* (60 years old male with CHF and diabetes)

HCPs also acknowledged the role that they played in facilitating self care of their patients. They acknowledged the discharge planner’s role in facilitating self management and worried that discharge planners had become hospital bed managers. HCPs recognised the need for self management models that have been tried and tested in Australia, [13] as noted by the National Health and Hospitals Reform Commission (NHHRC) final report [16].

### Insufficient holistic and continuing care

One of the most common challenges experienced by participants related to insufficient GP and specialist consultation time. Consultations concentrated on the immediate problem, leaving little time to discuss warning signs of emerging problems associated with the chronic illness. In response to these limitations, several patients and carers said that they wanted their HCPs to be more knowledgeable and understanding of their conditions and that the consultation not be limited to the current symptoms, but sensitive to their context. For example one said,

*A lot of doctors are really marred, as far as your attendance is concerned. If you go in with a sore throat, they don’t care if that leg is bleeding or is falling off, you know? That’s the impression you would get. But he* [patient’s GP] *wants to know why and he’s searching, you know… He looks below the surface, you know?* (80 year old male with diabetes and CHF)

HCPs also recognized the narrow focus of clinical encounters, and supported a more holistic approach to health care. They suggested that a shortage of medical practitioners meant that insufficient attention was given to co-morbidities.

Supported and holistic care is a solution to this problem. The HCPs suggested that inadequate workforce and specialisation contributed to this single illness focus of care.

### Lack of patients and carers involvement in decision-making

Patients and carers were asked if they felt involved in making decisions about their condition. Seven patients and family carers reported that HCPs did not readily trust or act on their knowledge of their signs, symptoms and beliefs about their illness. This limited the capacity of patients and carers to participate in their care because there was a perception that HCPs were dismissive and failed to take the views of patients into consideration.

*When they said you must wear these socks and something else, I couldn’t help saying, I would be crippled if I wore them, and then what do they come up with ..//.. Why aren’t I included in the talk, you know what I mean?* (75 years old male with diabetes)

HCPs agreed that the inclusion of patients in planning and decision making about their health could be very helpful in achieving a better health outcome. Limited time and human resources were seen as significant barriers.

## Discussion

This study has demonstrated that patients with chronic illnesses, who received care from a wide range of health services, faced a range of challenges in accessing these services and had serious concerns about their unmet needs and wants within the health system. These include poor communication with HCPs and provision of information, poor organisation of service delivery and long waiting time to see HCPs, insufficient facilitation of self care, insufficient holistic and continuing care and not involving patients and carers in decision-making. Most of these has been recognised by the NSW Severe Chronic Disease Management Program [17] and all are closely related to the key concept of patient-centred care.

These concerns feature strongly in international experience [11,18-20] and reflects experience in Australia too. We have interpreted these as relevant to the needs, wants and preferences of patients in the belief that these influence health services and the level of care that patients receive [21]. In their survey of 7505 patients, Barton et al., reported the degree to which patients’ [22] wants and needs were met in general practices throughout Australia (response rate 60%). According to their survey, 70% of participants felt that their needs and wants were met and that their care was very good or excellent [22]. In contrast, the present qualitative study demonstrated that patients and carers with chronic illnesses, who receive care from a wide range of health services, faced a range of challenges in utilizing those services and had serious concerns about their unmet needs and wants within the health system. This is consistent with the results of a qualitative study by NSW Health [23].

Given the frequent endorsement in policy rhetoric that patient centredness is a desirable attribute of a health care system that technically and humanely meets the needs of patients with chronic illness, it is reasonable to critically appraise these policies to assess whether the rhetoric translates into policy action.

The National Chronic Disease Strategy and National Service Improvement Frameworks are the current Australian national policy guides that reflect the journey of chronically ill patients from prevention to management [24]. However, they do not contain implementation plans since these have been left to State health authorities. While patient centredness has not been defined in these documents, patient needs have quite clearly been identified as the principal element in providing optimal care for chronically ill patients [25].

Currently, there are several chronic care programs running in NSW [17,26], ACT [27], and other Australian states [28-31], all of which are intended to address the challenges of caring for chronically ill patients and to assist them to better manage their conditions. According to the national strategy, these policies should contain implementation plans [24].

The NSW Chronic Care Program is the main NSW Health initiative aimed at reducing avoidable hospital admissions and improving quality of life for people with chronic illness and their carers [4]. Although the term patient centredness has not been mentioned in the first phase of the NSW Chronic Care Program, the concept has been introduced as the first principle of the program in its second phase [32,33]. Patient centredness has not been defined clearly in any of the program’s relevant documents, but rather it has been taken to mean “placing patients at the centre of care [which] has implications for what, how, where and when care is delivered” [32]. The aim has been mentioned in order to have an impact on the health services delivered locally so that it is more responsive to local and individual patient needs [32].

As part of the Perfecting Health Care Delivery Initiative (Maggie Program) of Hunter New England Area Health Service, a qualitative study was conducted by NSW Health to investigate the concerns of patients and carers in receiving services from the health system. The main issues identified were: a) difficulties and delays in accessing services and frequent cancellation of appointments; b) poor communication from staff; c) long waiting times once patients have arrived for an appointment and overbooking of clinics; d) busy, noisy environments where staff did not seem to know what they were doing; and e) patients having to move to a number of different places to receive treatment [34]. These findings are confirmed by those of the present study. However, these have not been reflected in the NSW Chronic Care Program as performance goals. Rather, to ensure that the program remains truly patient focused, it was suggested that working parties refer continually to the question, “What is best for the patient?” [34]. This approach does not provide sufficient direction about how health systems can best address the needs and wants of patients.

Similarly, in the ACT, the first principle of the ACT Chronic Disease Strategy 2008-2011 is patient centredness [35]. It is defined as “care where people are consulted on all aspects of their care, and care is focused on the whole person, care is planned in partnership with the patient and their carers and family as agreed with the person or their advocates” [35]. While this definition is clearer than that in the NSW Chronic Care Program, it does not mention patients’ wants and preferences [36]. Furthermore, there is no clear implementation plan to achieve the goal of patient centred care in the draft program which was published in 2008. Potentially, the ACT definition of patient centredness can be used for resource allocation and goal setting to a greater degree than the NSW Chronic Care Program.

Although there are examples of implemented patient centred programs in NSW [34,37], there is no strategic or business plan available on NSW Health, SWAHS or ACT Health websites which guides action in the health system. This may be due to unclear definitions of patient centredness in the chronic care programs.

Despite this gap, a number of patient centred outcomes have been used as measures to identify the successful implementation of strategies to evaluate various models of care in NSW [38]. These include improved documentation of patient encounters, reduced numbers of falls and medication errors, reduced numbers of re-presentations to emergency departments and re-admissions, increased numbers of attendance at health promotion activities and primary health care facilities, improvements in a range of mental health outcomes and generic measures of improvements such as reduced numbers of complaints and critical incidents [38]. Each of these factors is considered to be an essential ingredient of a health system that is focused on the overall health and well-being of patients. Interestingly, patients’ needs, wants and preferences are missing from the list.

Access to holistic patient centred care with consumers as partners has been identified as one of many frequently raised issues and suggestions to reform primary health care by community groups in the recent NHHRC final report [16]. At a state level, the concept of patient-centredness has also been identified by the Special Commission of Inquiry into Acute Care Services in NSW Public Hospitals as an essential ingredient in the reform of the NSW hospital system [39]. The report says that effective care for people with chronic illness must be based on the needs of patients rather than on the management of specific medical conditions, an approach that has been confirmed by the people we spoke to during the course of this study.

The authors of this paper consider that answering the question, “What is best for the patient?” [34] will not be sufficient to achieve a patient centred health system which is the first principle of both NSW and ACT chronic care programs [33,35,40]. Instead, we argue in this paper that needs, wants and preferences of patients and carers should be found and addressed in the chronic care programs.

As this paper has shown, patient centredness has been placed rhetorically at the centre of national and NSW chronic disease programs. However, there is neither a clear definition of patient centredness nor are there clear performance goals, implementation plan, and evaluation criteria in place. Findings from this study have confirmed the importance of integrating the needs and wants of patients into chronic care programs. As HCPs have indicated, close attention can be paid to these needs and wants of patients by improving patient health literacy, improving communication with HCPs, enhancing access to information technology and addressing other communication related issues.

This study has clearly identified what patients and carers want from the health system and these are similar to those identified by NSW Health [34]. These can be used to set up strategies and implementation plans with key performance areas to achieve patient centredness. These must then be translated into health system redesign features to address the unmet goal of the NSW Chronic Care Program. Ideally, this needs to be replicated in ACT and across Australia.

The finding of this study may be limited by the fact that they are based on interviews conducted in two specific locations in Australia and as a result, they may not be generalisable to other locations. The transcriber was unable to identify individual HCPs so it was not possible to ascribe comments to specific people or to recognise their organisation.

## Conclusion

Although patient centredness is the main stated principle of national, ACT and NSW chronic care programs, it has not been clearly defined and there is no subsequent strategic or business plan in any of these jurisdictions that takes the principle and turns it into actions that can be easily implemented. Furthermore, no specific resources have been allocated to the goal of achieving patient centredness.

If patient centredness is to have meaning as a principle within policies for the care of chronically ill patients, it must first be clearly defined. Second, the detail of patient centredness for various patient conditions must be analysed in sufficient depth to enable necessary and relevant actions to be defined. Finally, this process will allow those actions to be examined in the context of institutional and community based clinical practice, accountability, financing and management with this having the potential to contribute significantly in overcoming challenges posed by chronic illness.

## Endnote

^a^Pharmacists were recruited to the larger study but they have been excluded from this analysis because of the focus on patient experience with health services and health care professionals.

## Competing interests

The authors declare that they have no competing interests.

## Authors’ contributions

MM led the drafting of the manuscript. CA made a substantial contribution to the writing of the paper. MM, Y-HJ, BE designed and implemented the qualitative study, and participated in the recruitment, interviewing and analysis of data. SRL, PD and TU participated in the design of the main study (SCIPPS) and supervised the qualitative study. All authors contributed to the writing and review of the manuscript and all authors have approved the final manuscript.

## Pre-publication history

The pre-publication history for this paper can be accessed here:

http://www.biomedcentral.com/1472-6963/13/251/prepub
